# Intravitreal triamcinolone with transpupillary therapy for subfoveal choroidal neovascularization in age related macular degeneration. A randomized controlled pilot study [ISRCTN74123635]

**DOI:** 10.1186/1471-2415-5-27

**Published:** 2005-11-25

**Authors:** Ricardo Agurto-Rivera, Jose Diaz-Rubio, Luis Torres-Bernal, Tamer A Macky, Juner Colina-Luquez, Gabriela Papa-Oliva, Rama D Jager, Susana Martinez-Jardon, Jans Fromow-Guerra, Hugo Quiroz-Mercado

**Affiliations:** 1 Retina Service, Asociación Para Evitar la Ceguera (APEC), Mexico City, Mexico; 2 Department of Ophthalmology, Kasr El Aini Hospital, Cairo University, Cairo, Egypt; 3 Department of Ophthalmology, Harvard Medical School, Boston, Massachusetts, USA

## Abstract

**Background:**

To assess the effect of intravitreal triamcinolone acetonide (iTA) as an adjunctive treatment to transpupillary therapy (TTT) for new subfoveal choroidal neovascular membranes (CNV) in age-related macular degeneration (AMD).

**Methods:**

This prospective randomized controlled pilot study comprised 26 patients scheduled to receive TTT, due to either absent indications for photodynamic therapy or financial issues. Patients were assigned into; Group A (n = 14) received TTT alone and Group B (n = 12) received iTA (4 mg) followed by TTT within one week. Follow ups were at 2 weeks, and 1, 3 and 6 months for; best-corrected visual acuity (BCVA) by ETDRS chart at 4 meters, intraocular pressures (IOP), fluorescein angiography (FAG), and central foveal thickness by optical coherence tomography (OCT).

**Results:**

All 26 patients completed 6 months of follow ups. The average age for both groups was 74 years. Occult CNV formed 64% and 41%; classis/predominately classic 21% and 16.6%; and minimally classic 15% and 42.4% of group A and B respectively. At baseline; the mean BCVA was 0.045 for group A and 0.04 for group B; mean CNV size was 6.15 disc diameter (DD) and 2.44 DD; mean OCT foveal thickness was 513 um and 411 um for group A and B respectively with no statistical differences (P = 0.8, 0.07, and 0.19). At six months the proportion of patients gained ≥ 1 lines was 14% and 25% (P = 0.136) and stabilization was 86% and 66% (P = 0.336); the mean size of the CNV was 5.63 DD and 2.67 DD (P = 0.162); rate of CNV closure was 64% and 83% (P = 0.275); and the mean OCT central foveal thickness was 516.36 um and 453.67 um (P = 0.341), for group A and B respectively.

**Conclusion:**

The use of iTA as an adjunctive to TTT for new subfoveal CNV in AMD showed a tendency towards better functional results. However due to the small sample size of the study a statistically significant results could not be reached.

## Background

Age-related macular degeneration (AMD) is one of the leading causes of blindness in the western world; with the most common cause of visual loss is the formation of choroidal neovascularization (CNV) [1]. Laser photocoagulation has been proven to be more effective than the natural history of the disease process in both extra- and juxtafoveal CNV [2,3]. Currently, photodynamic therapy (PDT) with verteporfin and laser photocoagulation are the only proven therapies for the subfoveal CNV [4-8].

PDT has been proven beneficial for patients with both predominately classic CNV and with some benefits for occult with no classic CNV [4,6]. A retrospective review of 1000 consecutive patients with CNV in AMD showed that 17.1% had predominantly classic CNV [9]. In addition, analysis has shown that the treatment has minimal cost effectiveness, which is principally due to the high cost of the drug, the need of many retreatments, and the continuing visual decline that most patients experience even with re-treatments [10].

Transpupillary therapy (TTT) is a technique in which heat is delivered to the choroid and retinal pigment epithelium through the pupil using an 810-nm infrared diode laser. The diode laser has theoretical advantages over other wavelengths of light because there is little absorption in the xanthophyll layer and thus damage to the nerve fiber layer is minimized. Also, it is poorly absorbed by hemoglobin allowing an improved ability to treat through preretinal and subretinal hemorrhage [11]. The wavelength of the diode laser is mainly absorbed by melanin at the level of the choroid and retinal pigment epithelium, enabling treatment of choroidal lesions [12]. TTT is also significantly less expensive than PDT. It has been used to treat choroidal melanomas, and in preliminary trials to treat both classic and occult subfoveal CNV [13-17].

There is evidence suggesting that steroids may have a beneficial effect in patients with CNV. Eyes with CNV have histopathologic evidence of inflammation, and neovascularization is a frequent component of inflammatory processes [18-20]. Histopathological examination of CNV complexes has shown the presence of inflammatory cells [18-20]. In addition the amount of vascular endothelial growth factor (VEGF), the major cytokines involved in initiating angiogenesis, has been shown to be proportional to the amount of inflammatory cells present [21]. Corticosteroids also have in addition a direct antiangiogenic effects [22-25]. Triamcinolone acetonide and other steroids have been shown to be effective in inhibiting neovascularization in animal models [26,27]. Several clinical studies have shown an apparent beneficial effect where treated patients appeared to have a favorable effect on visual acuity and fundus appearance, although a significant proportion of patients still lost vision [28-30].

It may be possible to treat patients with CNV with TTT plus iTA, to combine the immediate effect of TTT with the longer-lasting, anti-inflammatory and possibly synergistic effect of intraocular triamcinolone. To help investigate this possibility we started a randomized controlled pilot study of combined TTT with iTA for CNV in patients with AMD.

## Methods

Although a randomized, multicenter, prospective, placebo-controlled trial (TTT4CNV, preliminary results, ARVO 2005) is underway to investigate the value of TTT in the treatment of occult subfoveal CNV, we did not find any report in a medline search that evaluates the effect of intravitreal triamcinolone with TTT for subfoveal CNV. Considering recent reports that demonstrate beneficial effects when triamcinolone is associated to PDT, we decided to conduct a prospective randomized non-masked clinical study of combined TTT with iTA in patients with CNV secondary to AMD.

Approval for the study was obtained from the hospital's ethical committee which is in compliance with the Helsinki Declaration. All patients received a thorough explanation of the study design and aims, and were provided with written informed consent. Patients were seen at Asociación Para Evitar la Ceguera en Mexico, "Dr. Luis Sanchez Bulnes Hospital", Mexico City, Mexico. All patients had a baseline evaluation for the following:

- Best-Corrected Visual Acuity (BCVA) that was evaluated using an ETDRS chart, measured with refraction obtained at the beginning of the study and recorded as a decimal equivalent value. Special careful was taken to avoid extrafoveal fixation.

- Slit-lamp biomicroscopy

- Indirect ophthalmoscopy

- Flourescein angiography (FAG)

- Optical Coherence Tomography (OCT) measurement of central foveal thickness.

Eligibility criteria includes: 55 years or older, new CNV under the geometeric center of the fovea, and VA of <0.20. No restriction to the type of the membrane (classic, predominately classic, or occult) was made. The patient had to have a clear media and the ability and willingness to understand the informed consent. Patients were excluded if they had received previous treatment, have any condition other than AMD to account for the CNV or refuse follow up. Patients could not have pre-existing atrophy of the fovea or a rip of the retinal pigment epithelium (RPE). Patients were also excluded if they were using corticosteroids. They could not have any disease that would interefere with the treatment, increased risk of side effects, or been confused with side effects of the treatment (as anticoagulant treatment, hepatitis, prophyria, uncontrolled glaucoma, or sensitivity ot the drug or the flourescein dye used in the trial). Only one eye per patient was entered in the study.

Once the patients accepted to be part of the study, were randomly assigned by an unmasked investigator (RA) using a random number table, to one of two groups: **Group A **received transpupillary thermotherapy (TTT) alone, and **Group B **received TTT within a week of intravitreal triamcinolone acetonide injection (iTA). Same investigator (RA) treated the patients, knowing the assigned group before to apply TTT or inject iTA.

### Transpupillary therapy

Transpupillary thermotherapy was delivered through a slit lamp using a modified infrared diode laser at 810 nm with an adjustable beam width of 1.2 mm, 2.0 mm, 3.0 mm and 4.3 mm (Iris Medical Instruments, Mountain View, CA). The treatment parameter was adjusted according to the CNV type and size. Topical 0.5% proparacaine was applied before placement of a three mirror Goldmann lens coated for use with the diode laser. Continues observation through the slit lamp ensured fixation. Treatment was initiated with one spot for 60 seconds' duration at a power setting ranging between 360 and 880 mW such that no visible change or a barely detectable light-gray appearance to the lesion was present at the end of the treatment. Power settings was proportional to the spot size with larger spots requiring higher energy levels. In general, for a 2-mm spot size, the initial power level was between 360 mW and 700 mW. The spot size was adjusted to be 500 um larger than the membranes' greater diameter, if the CNV is larger than 4300 um, then overlapping spots were used. If any retinal whitening was observed or patient felt any pain, the power of the laser was decreased by 100 mW. Treatment was re-initiated and if retinal whitening continued to be observed, the power setting was again decreased by 100 mW. Care was taken to ensure that the entire lesion border was covered with treatment beam.

### Intravitreal triamcinolone injection

Intravitreal triamcinolone acetonide were given as follows: patients received several drops of topical proparacaine and one drop of Betadine 5% solution (Purdue Pharma, L.P., Stamford, CT). They were given then topical flouroquinolone drops once every 5 minutes for 30 minutes. A wire speculum was inserted in the eye. The patient reclined slightly and was instructed to look up. An injection of 0.1 cc triamcinolone (Kenalog 40 mg/ml, Bristol Myers Squibb, New York, NY) was given at the 6-o'clock postion 3.5 to 4 mm posterior to the limbus using a 27 gauge needle. During the injection the needle was inserted 3 to 4 mm into the eye. The intraocular pressure was measured 5 minutes afterwards. If the IOP exceeded 24 mmHg at any time during follow ups, patients were given topical medications. If IOP exceeds 40 mmHg an anterior chamber paracentesis using a 30-gauge needle to remove 0.1 cc of aqueous humor was performed.

### Follow ups

Patients were give topical antibiotics to use four times per day for one week. Patients were seen in follow ups at 2 weeks, and 1,3 and 6 months after the treament. At each follow up visit, they had BCVA measurement, slit lamp biomicroscopy, intraocular pressure measurement (IOP), and indirect ophthalmoloscopy. FAG, and OCT measurement of foveal center were done at 3 and 6 months. Retreatment was performed using the same protocol if there was no change in subretinal elevation by clinical examination and OCT, together with persistent leakage on FAG at 3 and 6 months.

### Outcome measures

Primary outcomes were BCVA, CNV size and rate of closure, and OCT measurements at the foveal center. Main secondary outcome was retreatment rates.

## Results

There were 26 eyes of 26 patients enrolled in our study. The mean age was 74 years for both groups with no statistical difference in the age of the patients or other demographic data (Table 1) between the two groups. Baseline mean BCVA were 0.045 and 0.04 in groups A and B respectively, with no statistical significant difference (P = 0.885 Mann-Whitney). In group A there were 64% (9/14) occult CNV, 21% (4/14) classic/predominately classic CNV, and 15% (1/14) minimally classic CNV. In group B there were 41% (5/12) occult CNV, 16.6% (2/12) classic/predominately classic CNV and 16.6% (2/12) minimally classic CNV, 3 patients was undetermined. The breakdown of the type of the CNV showed no statistical difference between both groups (P = 0.095 Mann-Whitney). The mean CNV size at baseline was 6.15 disc diameter (DD) and 2.44 DD for group A and B respectively (P = 0.075 Mann-Whitney). The mean OCT foveal thickness was 513 um and 411 um for group A and B respectively at baseline with no statistical significant different (P = 0.190 Mann-Whitney).

### Six-month data

The six months follow up was available for all 26 patients. Media BCVA was 0.045 for both groups. The proportion of patients gained ≥ 1 lines was 14% (1/14) and 25% (2/12) for group A and B respectively (P = 0.336 Wilcoxon test), and stabilized in 86% (12/14) and 66% (8/12) of patients in group A and B respectively (P = 0.26 Wilcoxon test) both of which were not statistically significant. The mean size of the CNV was 5.63 DD and 2.67 DD for patients in group A and B respectively (P = 0.162 Mann-Whitney). Rate of CNV closure was 64% (9/14) and 83% (10/12) for group A and B respectively at six months (P = 0.275 Chi Square). The proportion of patients with subretinal fluids was 60% and 50% with the mean OCT central foveal thickness was 516.36 um and 453.67 um, for groups A and B respectively (P = 0.341 Mann-Whitney). All the above mentioned data were not statistically significant when compared to baseline and between both groups.

Occult CNV had more lines gain, where 60% (3/5) of patients receiving iTA showed lines gain compared with 22% (2/9) of patients not receiving iTA. Also patients with occult CNV had a relatively better response to iTA than classic membranes where 66% of patients without iTA showed lines gain compared with 80% of patients with iTA. The retreatments rate was 36% and 17% for groups A and B respectively (P = 0.175 Chi Square).

### Complications

No patient had evidence of endophthalmitis at any time point. An increase in intraocular pressure beyond 23 mmHg was experienced in 2 patients in group B, who did not have previous glaucoma. Intraocular pressure was controlled with topical medication using beta blockers in both patients. Mean IOP at baseline was 15.1 and 16.3 mmHg, and at six months was 15.0 and 16.3 mmHg for groups A and B respectively. However two weeks after triamcinolone injection the mean IOP in group B was 17.9 mm Hg. The lens status was not graded in this study by a formalized method such as the Lens Opacities Classification System (LOCS II) [31]. However progression of nuclear sclerosis was not seen in any patient.

## Discussion

This single-center prospective comparative randomized pilot study examined the use of combined TTT with iTA for the treatment of CNV secondary to AMD. We found that although there was no statistical significant difference in the functional result between both groups there was a trend in favor of the combined TTT with iTA. Patients receiving iTA with TTT had more line gains at six months, than those that did not receive iTA. In addition there was less retreatment in patients in the iTA group, than in the other group. However the anatomical results were clinically and statistically insignificant, in terms of the final size of the CNV, and OCT central foveal thickness.

Although exudative AMD is the leading cause of visual loss in patients above 60 years in western countries, conventional PDT with verteporfin can be given as a treatment in a minority of those with the disease [1]. In addition patients with CNV require multiple retreatments. The proportion requiring retreatments at the first 3-month interval is 90.8% for the TAP study and 68.9% for the VIP study [4-6]. In addition, PDT has minimal cost effectiveness as shown by previous studies [10]. TTT on the other hand is significantly less expensive than PDT and its energy penetrate deep to the choroid and RPE with minimal absorption by neurosensorial retina. The energy is eliminated like heat causing an elevation of local temperature (15–20 degree Celsius) inducing apoptosis with thermal inhibition of angiogenesis and vascular thrombosis [14,15]. TTT in preliminary trials showed benefits in treating both classic and occult subfoveal CNV [14-17].

There are several possible reasons to combine TTT with iTA. Choroidal neovascularization have other constituents such as inflammatory cells, and other signs of inflammation that might not benefit from the short term angiogenic effect of TTT [18-20]. Corticosteroids have a direct antiangiogenic effects [22-25]. And have been shown to be effective in inhibiting neovascularization in animal models [26-28]. Steroids can modulate the production of and reduce the permeability increased by VEGF. These secondary effects would not be expected to occur with TTT alone. Intravitreal TA persists in the vitreous cavity which extends the duration of treatment against the neovascular complex [32,33]. Several clinical studies have shown an apparent beneficial effect where treated patients appeared to have a favorable effect on visual acuity and fundus appearance, although a significant proportion of patients still lost vision [29-31].

In his pilot study, Reichel et al had 16 patients with occult subfoveal CNV treated with TTT with 12 months of follow ups [14]. Visual acuity improved 2 or more lines in 19%, stabilized in 56%, the exudation decreased in 94% of patients, and 19% of cases had to be re-treated. Other authors described improvements of 2 or more lines between 12.4 to 30% of cases, and VA stabilization between 40 to 43%, exudation reduction was around 75% and approximately 25% of patients required re-treatment [15-19]. In our study we obtained 14% and 25% VA improvement of one or more lines for group A and B respectively. However the little number of patients did not permit us to establish a statistical difference between groups, but our results are comparable to those reported in the literature. Also results of stabilization of VA, which was 86% and 66.6% of patients in group A and B respectively, was consistent with previous reports. We believed that these results must to be considered in the context of patients with very low VA, so excessive diminution was difficult, also many patients were considered out of therapeutical limits due to big size of membrane and bad VA, even though we could obtain improvement from baseline visual acuity.

Complications arising from this treatment may be expected to include all of those that could occur from TTT therapy as well as the incremental risks posed by the intravitreal injection of triamcinolone. The additional risks are principally increased IOP, progression of cataract formation, and endophthalmitis. In the present study we have increased IOP in 2 patients in group B, both of which were controlled by medications and resolved completely in 3–4 months after treatment. Progression of cataract and endophthalmitis were not seen in our study group.

This study is limited by the small number of patients in each group with a limited follow ups. The physicians were not blinded during examination of the patients or FAG. The patients also knew they were in an experimental study. Therefore finding from this study should not be used as a justification to treat patients in an uncontrolled fashion. However in the course of development of new treatment strategies, the iteration starts with pilot series data that are used to formulate larger better controlled, but more expensive studies.

## Conclusion

The findings from this study suggest that the combination of transpupillary thermotherapy along with intravitreal triamcinolone offers the possibility of better functional results than with TTT alone, meriting additional randomized study.

## Competing interests

The author(s) declare that they have no competing interests.

## Authors' contributions

RAR, JDR, LTB, TAM, JCL, GPO, RDJ, SMJ, and JFG have made substantial contributions to the conception, design, and acquisition of data together with data analysis and interpretation, and were involved in drafting the article and revising it critically for important intellectual content. HQM has given his final approval for that version to be published.

## Pre-publication history

The pre-publication history for this paper can be accessed here:



## Figures and Tables

**Figure 1:**
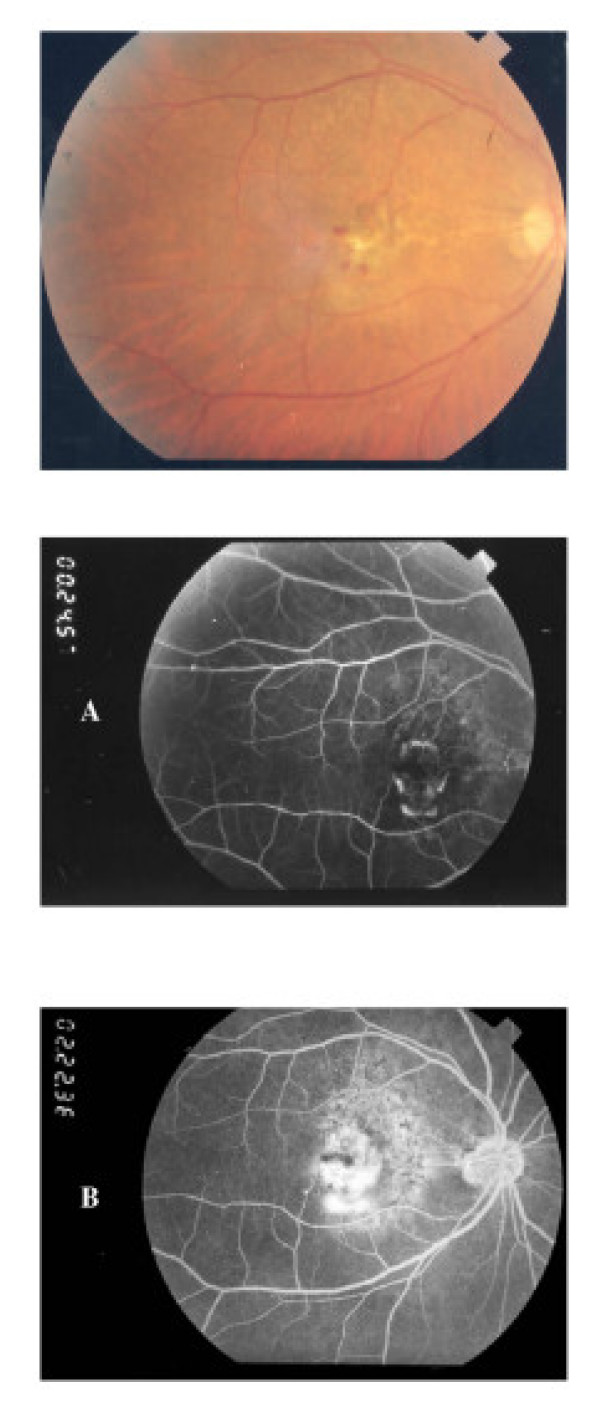
A 65 year old patient with visual acuity of 1/10 in right eye that developed visual lost three months earlier. Color photograph shows a CNV with a small hemorrhage and sub retinal fluid. Fluorescein angiogram shows **A: **Early RPE defects above and below the foveal center. Later **B: **shows late dye leakage indication occult CNV.

**Figure 2:**
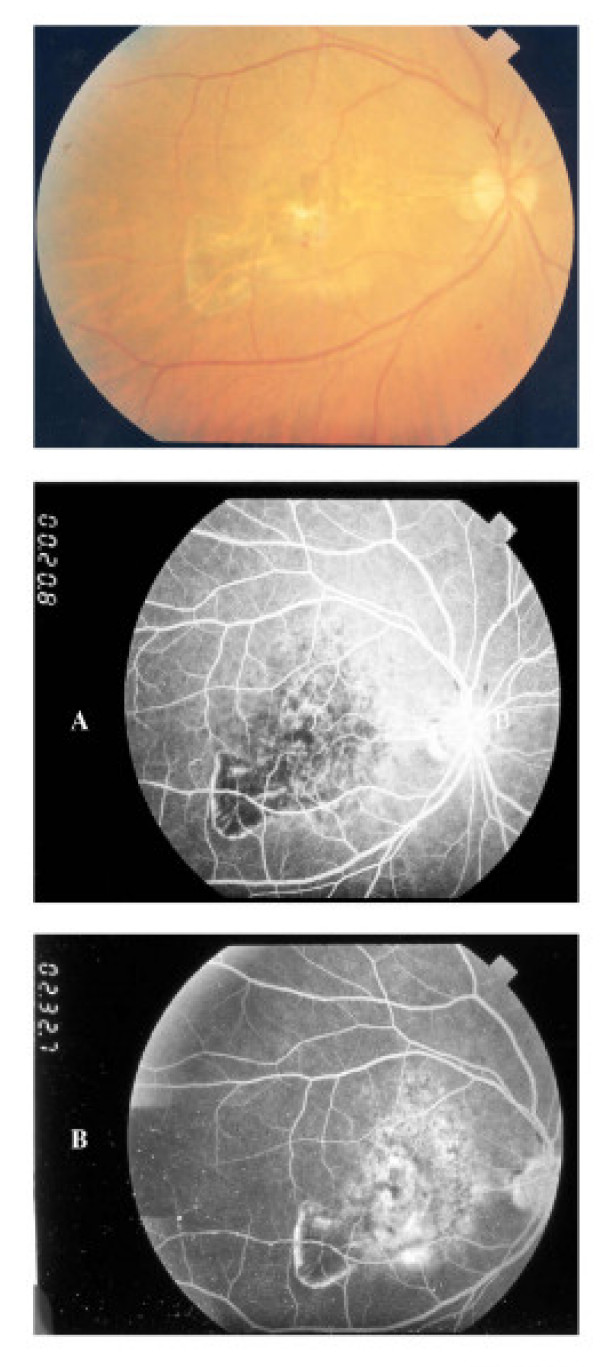
Patient in figure 1, four months after TTT and iTA. Color photograph shows absence of sub retinal fluid and hemorrhage. Fluorescein angiography shows in **(A) **a large area of RPE and choroidal atrophy with **(B) **late staining of the enlarged chorioretinal scar after treatment with no evidence of dye leakage.
